# The effect of diode laser irradiation associated with photoabsorbing agents containing remineralizing materials on microhardness, morphology and chemical structure of early enamel caries

**DOI:** 10.4317/jced.55059

**Published:** 2018-10-01

**Authors:** Farzaneh Ahrari, Hamideh-Sadat Mohammadipour, Ladan Hajimomenian, Amir Fallah-Rastegar

**Affiliations:** 1 Assistant Professor of Orthodontics, Dental Research Center, School of Dentistry, Mashhad University of Medical Sciences, Mashhad, Iran; 2 Assistant Professor of Restorative Dentistry, Department of Restorative and Cosmetic Dentistry, School of Dentistry, Mashhad University of Medical Sciences, Mashhad, Iran; 3 Dental Research Center, School of Dentistry, Mashhad University of Medical Sciences, Mashhad, Iran

## Abstract

**Background:**

This study investigated the effects of laser irradiation associated with photo-absorbing agents containing sodium fluoride (NaF), MI paste Plus or Remin Pro® on microhardness and surface structure of white spot lesions (WSLs).

**Material and Methods:**

Fifty-six premolars were divided into two halves, then immersed in a demineraling solution to induce WSLs. The samples were divided into 8 groups by treatment (n=12) : (1) control, (2) diode laser (810 nm, 500 mW, 90 s), (3) NaF, (4) MI Paste plus, (5) Remin Pro®, (6) NaF + Laser, (7) MI Paste Plus + Laser, (8) Remin Pro® + Laser. Microhardness was measured before and after remineralization treatments. Two samples from each group were selected for SEM analysis.

**Results:**

Microhardness increased significantly after all treatments with the exception of control, Laser and Remin Pro® groups (*p* >0.05). ANOVA revealed no significant difference in initial microhardness (*P*=0.21), whereas a significant difference was noted after treatment (*P*=0.009). The application of sodium fluoride with or without laser irradiation produced the highest microhardness among the groups (*p*<0.05). SEM analysis revealed some cracks on lased enamel and non-homogenous coatings of minerals after the use of remineralizing products.

**Conclusions:**

The use of NaF either alone or combined with laser irradiation was the most effecttive strategy for increasing microhardness of WSLs. The application of diode laser through photoabsorbing agents containing sodium fluoride or MI Paste Plus did not produce any additional effects in enhancing remineralization of WSLs, whereas the combined application of diode laser with Remin Pro® was effective.

** Key words:**CPP-ACP, Enamel caries, fluoride, Hydroxyapatite, Low level laser, Microhardness, Remineralization, casein phosphopeptide amorphous calcium phosphate.

## Introduction

The earliest manifestation of dental caries is the production of subsurface porosities which lead to a milky-white appearance on the tooth surface, defined as white spot lesions (WSs). These lesions are more frequently observed in patients who have poor dental hygiene or in those undergoing fixed orthodontic treatment. WSLs not only produce an unaesthetic and disturbing appearance, but also can progress to caries cavities on the tooth surface (1,2).

The WSLs can be remineralized before surface cavitations through diffusion of fluoride, calcium and phosphate ions from remineralizing agents into their structure (3). Other remineralizing products such as casein phosphopeptide amorphous calcium phosphate (CPP-ACP) and hydroxyapatite have also been proposed in recent years for the prevention and treatment of caries affected surfaces. MI Paste Plus (GC Corporation, Tokyo, Japan) is a commercial product that contains CPP-ACP and 900 ppm fluoride (CPP-ACPF). Remin Pro® (VOCO GmbH, Cuxhaven, Germany) is another remineralizing cream containing hydroxyapatite, fluoride and xylitol. Previous studies reported promising results on the use of products containing CPP-ACP or hydroxyapatite for caries management (4-8). Uysal *et al.* (9) indicated that the effectiveness of CPP-ACP in inhibiting caries around orthodontic brackets was comparable to sodium fluoride. Bilgin *et al.* (4) exhibited that Remin Pro® produced a greater remineralization effect on enamel WSLs compared to CPP-ACPF, sodium fluoride and fluoride varnish.

Laser irradiation has been applied in recent years for its possible strengthening effect on tooth structure. It has been indicated that high power lasers such as CO2 laser can increase enamel surface hardness and reduce its solubility (2,10,11). However, high power lasers should be applied consciously and with low energy level to preserve enamel integrity and minimize the creation of porosities on the tooth surface (12). Low-power red and near-infrared lasers appear to be a tempting alternative to high power lasers for caries inhibition (3). However, there are only a few studies regarding the use of low power lasers for prevention and management of dental caries (13-16).

To alter the composition or solubility of enamel, the laser energy must be strongly absorbed and converted into heat without damage to tooth structure or surrounding tissues (3). The application of photo-absorbing agents have been suggested before irradiation of low power lasers to accumulate laser energy and increase its thermal effects. On the other hand, it has been indicated that the application of topical mineral agents combined with laser irradiatin produces a synergistic effect on caries treatment and reduces enamel solubility and permeability (2,10,12). Therefore, the photo-absorbing creams can be mixed with remineralizing agents for better remineralizing effects.

To our knowledge, no study investigated the effect of photo-absorbing creams containing remineralizing agents followed by low level laser irradiation on microhardness of WSLs. Therefore, this *in vitro* study was conducted to evaluate the effect of low power laser irradiation associated with photo-absorbing creams containing 2% sodium fluoride, MI paste Plus or Remin Pro® on microhardness, surface structure and chemical composition of WSLs.

## Material and Methods

-Sample selection and preparation 

In this *in vitro* study, 56 sound premolars extracted for orthodontic reasons were selected. Remained soft tissues, calculus and plaque were removed by hand instruments, rubber cap and pumice slurry from the tooth surfaces. The cleaned teeth were examined under a stereomicroscope (Dino lite Pro, Anmo Electronics Corp, Taiwan) at 10 X magnification to discard those with caries, cracks and developmental and structural defects in enamel structure. The selected teeth were then stored in a 0.1% thymol solution at room temperature until the time of the experiment. For sample preparation, the root of the tooth was separated, and the crown was bisected in mesiodistal direction into two halves, using a slow-speed water-cooled diamond saw (CNC Machine, Nemo, Iran). Therefore, two blocks were obtained from each tooth, one from the buccal and the other one from the lingual surface. Afterwards, the blocks were embedded in epoxy resin, in such a way that the buccal/lingual enamel was positioned horizontally, in contact with the working table. A treatment window of 4 × 4 mm was then exposed on the surface of each specimen, using 400, 600 and 800 grit silicon carbide papers. Finally, a random number was given to each resin block.

To induce artificial white spot lesions (WSLs), the samples were stored individually for 10 weeks in plastic containers, filled with a demineralizing solution. This solution consisted of 50 mM acetic acid, 2.2 mM CaCl2, and 2.2 mM NaH2PO4 with pH adjusted at 4.8 using potassium hydroxide (KOH). The demineralizing solution was replaced every five days. The presence of WSLs was confirmed visually in all the specimens by a magnifier.

-Surface microhardness measurement

The specimens were rinsed with distilled water and air-dried for 30 s. The initial microhardness number of each sample was measured using a Vickers microhardness tester (Matsuzawa, Japan). The indentor of the device was placed perpendicular to the early enamel caries surface. A vertical load of 100 g was applied for 5 s, and the indentation length was photographed and measured using a microscope and computer software. The test was performed at three close areas on each specimen and the mean value was calculated.

-Surface treatments

The enamel blocks were randomly assigned to eight groups (n=12), according to the treatment applied on demineralized enamel:

Group1 (control): No treatment was performed and the samples were immersed in distilled water.

Group 2 (Laser): The specimens in this group were irradiated by an infrared gallium aluminum arsenide (GaAlAs) diode laser (wavelength 810 nm; ARC Laser GmbH, Nürnberg, Germany). The beam was delivered perpendicularly through a 300 µm fiber optic cable at continuous-wave mode for 90 seconds. The power was set at 500 mW and the laser irradiation was performed with a light contact and sweeping motion to ensure covering the whole surface of the window.

Group 3 (NaF): A 2% sodium fluoride gel (Sultan Health Care Inc., Englewood, New Jersey, USA) was applied on enamel surface and remained for 5 minutes; then thoroughly rinsed with running water. The specimens were then stored in distilled water.

Group 4 (MI Paste Plus): The treatment windows were covered with a sufficient amount of MI Paste Plus (Recaldent, GC, Japan) containing CPP-ACPF for 5 minutes. The specimens were then rinsed and stored in distilled water.

Group 5 (Remin Pro®): A commercial cream containing hydroxyapatite and fluoride (Remin Pro®; VOCO, Germany) was applied on the enamel surface and remained for 5 minutes; then thoroughly rinsed with running water and the specimens were immersed in distilled water.

Group 6 (NaF + Laser): A photo-absorbing NaF agent was prepared by adding a blue eatable dye to NaF gel to increase laser absorption. The gel was placed on the enamel surface and the diode laser was irradiated immediately for 90 seconds through the gel using the parameters described beforehand. After 5 minutes of applying the photo-absorbing gel, the specimens were rinsed and immersed in distilled water.

Group 7 (MI Paste Plus + Laser): A photo-absorbing cream containing CPP-ACPF was prepared by adding a blue eatable dye to MI Paste Plus. The cream was placed on the enamel surface and the treatment sequence was followed similar to group 6.

Group 8 (Remin Pro® + Laser): A photo-absorbing agent containing hydroxyapatite and fluoride was made by adding a blue eatable dye to Remin Pro®. The cream was then placed on the enamel surface and the treatment sequence was continued similar to group 6.

Afterwards, the specimens were again subjected to microhardness assessment. The test was performed under the same conditions as described beforehand, and the mean value of three measurements was recorded for each specimen.

-Scanning Electron Microscopy (SEM) and Energy Dispersing X-ray (EDX) analysis

Two samples from each group were randomly selected to assess the surface morphology and chemical alteration of enamel after different surface treatments.

The surface morphology of the specimens was analyzed using a scanning electron microscope (SEM, VEGA II LSH, TESCAN, Czech Republic) at ×1000 magnifications. The samples were desiccated using 100% alcohol, and then coated with a thin film of gold for SEM examination.

The chemical composition of the specimens was evaluated using an EDX detector that was applied by an energy dispersion X-ray spectrophotometer (QUANTAX QX2, BRUKER/ROENTEC, Germany). The calcium, phosphate and fluoride contents were recorded in weight percent of the specimen.

-Statistical analysis

The normal distribution of the data was confirmed by the Kolmogorov-Smirnov test. A paired sample t-test was applied to determine any significant alteration in microhardness values before and after treatment in each of the study groups. One way analysis of variance (ANOVA) was run to detect any significant difference in microhardness values among the study groups either before or after treatment. When a significant difference was noted, pairwise comparisons were made by Duncan pot hoc test. The data were analyzed through SPSS software (Statistical Package for Social Sciences, Version 16.0, SPSS Inc, Chicago, Ill, USA) and the significance level was determined at *p*<0.05.

## Results

The results obtained from the paired sample t-test indicated that microhardness values increased significantly after all treatments with the exception of control (group 1), Laser (group 2) and Remin Pro® (group 6) groups, which showed no significant difference after treatment (*P*>0.05).

ANOVA revealed no significant difference in initial microhardness values among the study groups (*P*=0.21), but a significant difference was noted after treatment (*P*=0.009). Pairwise comparisons by Duncan test indicated that the application of sodium fluoride either alone or in combination with diode laser irradiation produced the highest microhardness value among the study groups (*p*<0.05;  Table 1).

**Table 1:**
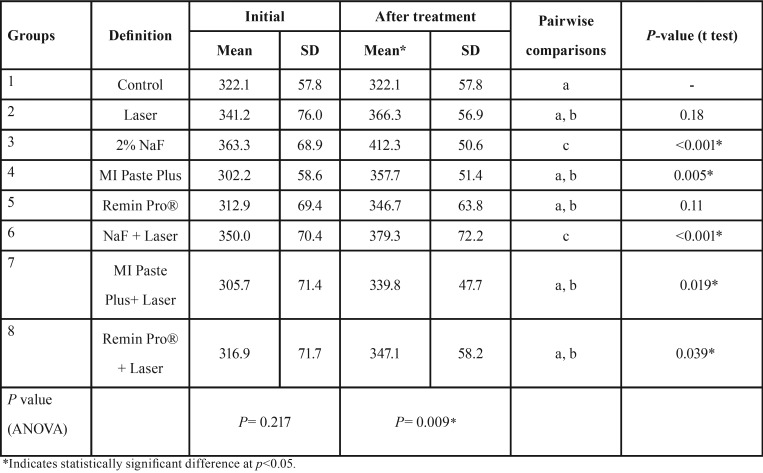
The mean, standard deviation (SD) and the results of the statistical analysis regarding microhardness values before and after treatment in the experimental groups.

-SEM analysis

**Figure 1:**
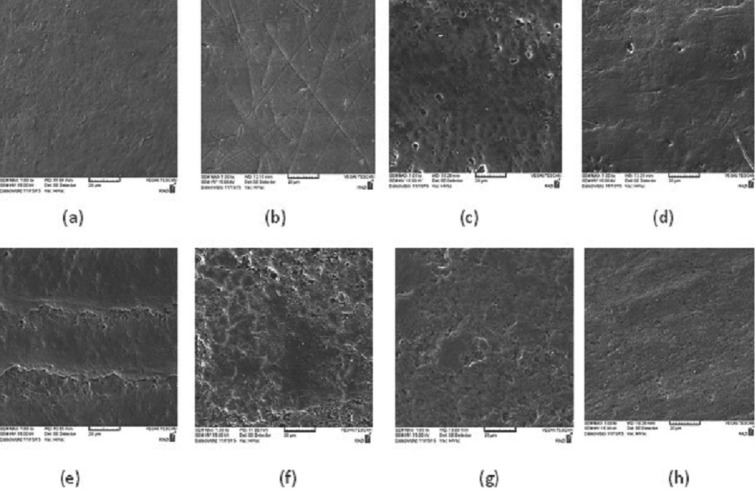
SEM images of the representative specimens in the study groups after different surface treatments: (a) control, (b) Laser, (c) 2% NaF, (d) MI Paste Plus, (e) Remin Pro®, (f) NaF + Laser (g) MI Paste Plus + Laser (h) Remin Pro® +Laser (SEM magnification 1.00 Kx).

In the control group, the natural appearance of enamel rods appeared to be faded away because of immersion in the demineralizing solution, which leads to the dissolution of mineral components (Fig. 1a). In laser treated samples, the morphology of enamel was obviously different from the control group, because there were cracks, scratches, and porosities on the surface (Fig. 1b). When the remineralizing agents were applied on the surface without laser irradiation, some non-homogenous coatings with numerous granular or globular patterns masked the underlying enamel surface (Fig. 1c-e). The granular pattern of minerals was more obvious in the specimens treated with NaF. In the groups which were treated with the combination of remineralizing agents and laser irradiation, a more obvious appearance of calcium phosphate in the form of separated or adherent granules was seen along with a lower degree of enamel cracks produced by the laser (Fig. 1f-h).

-EDX analysis

The mean values of minerals obtained from the two samples in the study groups are presented in  Table 2. The fluorine content was greater in MI Paste Plus and Remin Pro groups, and the Phosphorus content was greater in Remin Pro® groups.

**Table 2:**
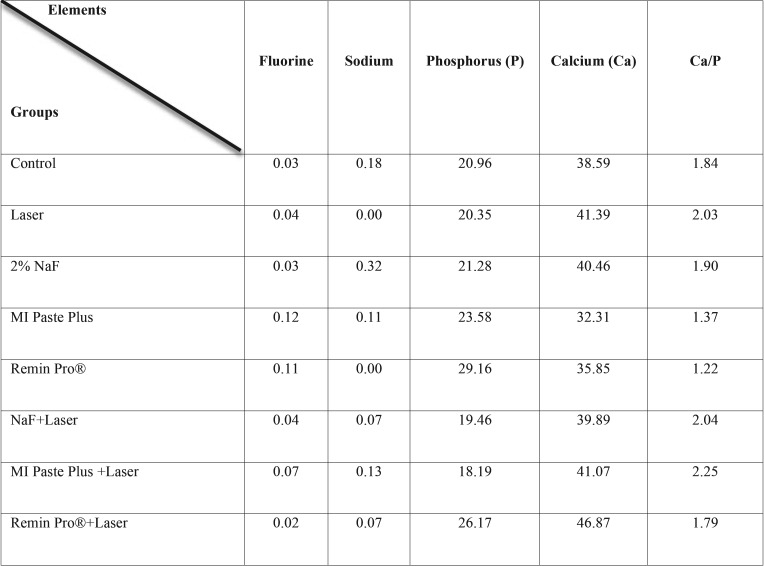
Comparison of some minerals (weight %) in the study groups.

## Discussion

The present study investigated the effect of different remineralizing agents either alone or combined with diode laser irradiation on microhardness of initial caries lesions. The surface microhardness test is generally proposed to assess the remineralizing efficacy of mineral agents through measuring surface strength (9). In the present study, microhardness assessment was performed in two stages; the first one was taken after tooth immersion in the demineralizing solution and the second one was obtained after the application of different remineralizing protocols. The lack of significant difference between groups after the first microhardness measurement confirms the presence of comparable enamel demineralization in the study groups. However, a significant difference in microhardness was observed after treatment among the groups. The application of NaF either alone or combined with laser irradiation was the most effective strategy to increase microhardness of WSLs. When the initial and final microharness values were compared in each study group, it was revealed that all remineralizing treatments caused a significant increase in microhardness, with the exception of Laser and Remin Pro® groups, which showed no significant difference after treatment.

In the present study, the application of an 810 nm diode laser at low power mode was not effective in enhancing microharness of WSLs. Previous studies generally used high power lasers such as CO2, Nd:YAG or Er:YAG lasers to enhance enamel resistance against caries attack (2,8-9,12,17-18). Different mechanisms have been proposed for caries-preventive effects of lasers. Some authors believe that laser irradiation leads to melting, fusion and resolidifaction of enamel crystals. Others assume that the reduction of water and carbonate content of enamel, the increase in hydroxyl ion content and the decomposition of enamel proteins are responsible for enhanced resistance against acid challenges (17,19). The laser power used in this study was 500 mW, which is the borderline between high power and low power lasers. Since the 810 nm diode laser has a low absorption band in tooth enamel, a photo-absorbing cream containing mineral agents was applied in this study before laser irradiation to enhance photochemical reactions and increase laser absorption by tooth structures. In some studies, (3,14,20,21) Indocyanine green (ICG) was applied as a photo-absorbing agent combined with the infrared diode laser. In this study, a blue eatable dye was employed because it was very cheap and more accessible than ICG. SEM analysis revealed surface cracks and scratches produced as a result of laser irradiation on enamel surfaces. Since WSLs have a demineralized and weak surface, the effect of laser on creating cracks may be strengthened compared to healthy enamel. The alteration in laser parameters such as wavelength, power, pulse frequency and duration of irradiation may lead to different results, so further studies are suggested. The outcomes of this study are in agreement with those of Kato *et al.* (16) who reported that diode laser irradiation (960 nm, 10 Hz, 6.5 W, 33 mJ) promoted a slight increase in calcium solubility. In contrast, De Sant’Anna *et al.* (3) revealed that the irradiation from an infrared diode laser (λ = 810 nm, 30 W) on deciduous tooth enamel using a photo-absorbing agent containing 2% sodium fluoride created reservoirs of minerals, which provided enamel protection against acid challenge during cariogenic activity phases.

In the present study, the groups treated with 2% sodium fluoride either alone or combined with laser irradiation presented the highest microhardness among the study groups. Although the caries preventive effect of fluoride-containing products has been largely described and understood, (5,9,22,23) the anticariogenic potential of laser irradiation combined with fluoride treatment still remains controversial (2,10,18,19,24-26). Several types of lasers have been used in combination with fluoride for prevention of enamel demineralization. Some authors believe that laser irradiation can increase binding of fluoride to tooth structure and lead to a significantly higher fluoride content in enamel (27). Others assume that the thermal effects of laser make several surface cracks which trap tooth minerals and fluoride ion in laser-irradiated substrate during a cariogenic challenge (11). However, the results obtained from this investigation showed that the diode laser irradiation through the photo-absorbing cream containing 2% sodium fluoride was not more effective than 2% sodium fluoride alone for enhancing microhardness of WSLs. This means that, laser irradiation did not show a synergistic effect with NaF on remineralization of incipient caries. The outcomes of this study are in line with those of Santaella *et al.* (28) who revealed that the fluoride varnish was more effective than diode laser application in enhancing the resistance of sound enamel. Similarly, Bahrololoomi *et al.* (13) revealed that the combined application of diode laser and topical fluoride varnish had no significant additional effect on enamel resistance to caries compared to fluoride varnish alone. Kato *et al.* (16) found that the additional application of 960-nm diode laser to the fluoride treatment caused no significant increase or decrease in calcium solubility.

The use of CPP-ACP in recent years is growing as a useful method for prevention and treatment of enamel caries (8). It has been shown that CPP-ACP can stabilize calcium phosphate in dental plaque in the proximity of the teeth. In the presence of acid challenge, such as after each meal, CPP-ACP helps to maintain a supersaturated state of minerals with respect to tooth enamel and thus restricts mineral loss and assists remineralization of enamel subsurface lesions (22). MI Paste Plus consists of CPP-ACP and 900 ppm fluoride. It is believed that the addition of fluoride into CPP-ACP enhances its remineralization potential on initial enamel lesions in comparison with either CPP-ACP or sodium fluoride alone (8). In the present study, the application of MI Paste Plus either alone or combined with laser irradiation caused a significant increase in microhardness of WSLs. But, the addition of laser irradiation to CPP-ACPF did not produce a synergistic effect in enhancing microhardness of WSLs.

Several studies confirmed the effectiveness of CPP-ACP containing agents in enamel remineralization. Salehzadeh *et al.* (7) found that the remineralization potential of MI Paste was significantly greater than sodium fluoride varnish and Remin Pro®. However, some studies presented no significant difference in inhibition of enamel caries between artificial saliva and MI Paste Plus (11,22). The authors attributed the results to the short treatment application time (only two minutes) and immediately rinsing all remnants of the products after treatment (11,22). The outcomes of this study confirm the results of Heravi *et al.* (15) who found that the combination of low power red and infra red lasers with CPP-ACPF provided no significant improvement in remineralization of enamel caries in comparison with CPP-ACPF alone.

In the present study, microhardness of WSLs increased after the application of Remin Pro®, but the increase was not significant. Remin Pro® is a water-based cream that contains three components: hydroxyapatite, 1450 ppm of fluoride and xylitol as a sugar substitute with cariostatic properties. It is assumed that the ingredients in this topical agent enhance the potential of remineralization; limit the adhesion of bacterial plaque to enamel and reinforce the tooth against further carious attack (30). The outcomes of this study are similar to those of Mohan *et al.* (31) who showed an increase in mineral content when CO2 laser irradiation was done before and after the application of Remin Pro® paste on the enamel surface. The findings of this study are in contrast to the studies that reported the products containing fluoride and hydroxyapatite showed a higher remineralization effect than commercial products containing only fluoride (1000 and 1450 ppm) (5) or MI Paste Plus (4).

When Remin Pro was associated with laser irradiation, a significant improvement in microhardness of WSLs was obtained. This may be related to enamel ultrastructural changes that were induced by laser irradiation. SEM images indicated that after laser irradiation some micro cracks were produced on the enamel surface which could enhance mineral penetration into the crystalline structure of enamel and change the superficially loosely bonded minerals into firmly bounded ones.

According to SEM images, the laser-treated surfaces presented fissures, cracks, and more roughness compared to the control group. These surface defects are assumed to trap and precipitate mineral agents that are present near the tooth structure during a cariogenic attack. On the other hand, these surface defects can weaken the tooth structure and serve as a reservoir for bacterial adhesion. The application of mineral-containing agents on the surface leads to globular patterns of minerals, which were more obvious in NaF group. It seems that these surface coatings are able to provide a certain degree of protection against cariogenic challenge during mineral loss phase. When laser irradiation was combined with remineralizing agents, the detrimental effects of laser on the surface diminished. De Sant’Anna *et al.* (3) reported that after treatment of sound deciduous teeth with infrared diode laser through a photo-absorbing agent containing 2% NaF, a homogenous surface coating that masked typical enamel surface was observed. A similar appearance was obtained in the present SEM analysis in the groups treated with remineralizing agents. However, the combined application of diode laser and remineralizing agents lead to more protruded and well-defined granular or globular irregularities with opaque borders on the surface. It is believed that the immobilization of calcium, phosphate, and fluoride on the lased enamel produces these morphological features.

The EDX analysis revealed that the fluorine content was greater in MI Paste Plus and Remin Pro® groups, and the phosphorus content was greater in both Remin Pro® groups. Due to devoting only two samples for EDX analysis, it was not possible to achieve a clear conclusion regarding the percentage of minerals after different surface treatments. In the study of Mohan *et al.* (31) an increase in Ca/p ratio was observed with the combination of laser irradiation and Remin Pro®. This was attributed to the presence of hydroxyapatite crystals in this topical paste (30).

The association between remineralizing agents and laser irradiation can be a subject of interest in future studies. This *in vitro* study had certain limitations such as difficulty in simulating oral environment with the same remineralization and demineralization cycles, the salivary flow rate and oral protective factors including salivary proteins and bacteria. Further studies should be conducted to investigate the combination of other remineralizing agents with different laser settings in the clinical situations.

## Conclusions

Under the conditions used in this study,

1. The application of NaF either alone or combined with laser irradiation was the most effective strategy for enhancing microhardness of WSLs.

2. The application of diode laser through photo-absorbing agents containing sodium fluoride or MI Paste Plus did not produce any additional effects in enhancing remineralization of WSLs

Although the combined application of diode laser with Remin Pro® was effective in enhancing microhardness of WSLs.
